# An Adaptive Allelic Series Featuring Complex Gene Rearrangements

**DOI:** 10.1371/journal.pgen.1002347

**Published:** 2011-10-20

**Authors:** Joshua M. Schmidt, Charles Robin

**Affiliations:** Genetics Department, The University of Melbourne, Melbourne, Australia; University of California Davis, United States of America

An intriguing observation from some studies of adaptive change is allelic series, where adaptive alleles successively replace each other at a single locus. For instance, at the *Cyp6g1* locus of *Drosophila melanogaster*, transposable element insertions and a gene duplication event have combined to create at least two adaptive alleles in which the more derived the allele, the greater the insecticide resistance of its bearer [1]. Similarly, insecticide-resistant alleles in *Culex* mosquitoes have been observed replacing each other within the period of a decade [2].

Another case of an allelic series is presented in the paper by Magwire et al. [3], which identifies a new locus affecting sigma virus resistance in *D. melanogaster*. Multiple alleles exist at this locus and they differ in their extent of gene copy number polymorphism and feature a transposable element thought to generate novel transcripts. Thus, this study contributes to an emerging picture that the mutations associated with recent adaptive events may not involve regulatory SNPs or coding SNPs, but complex gene rearrangements [1], [4], [5]. Furthermore, the nested nature of these rearrangements means that the order in which they arose can be deduced.

The genes featured in the particular rearrangement described by Magwire etal. [3] were originally identified via a novel genome-wide screen to identify transposable element insertions at high frequencies in natural populations [6]. Unlike the situation in humans and many other vertebrates, particular transposable element insertions are rarely at high frequencies in *Drosophila* populations. A survey of insertion site occupancy led Aminetzach and colleagues [6] to a gene, which they dubbed *CHKov1*, that has a *DOC* transposable element inserted into the coding region. This gene is one of a large cluster of 27 paralogs that encode proteins with distant similarity to choline kinases. The pattern of polymorphism around the *DOC* insertion suggests it was at the center of a very recent and strong selective sweep dating to between 25 and 240 years ago. What selective agent could result in such strong selection on an insect species, so recently? The link to “choline” motivated Aminetzach et al. [6] to test whether a commonly used class of insecticides, the organophosphates (OPs), which target the insect nervous system by inhibiting the enzyme acetylcholine esterase, could be the selective agent driving this selective sweep at a locus implied in choline metabolism. They found that a line bearing the *DOC* allele had greater resistance to an OP than a control line with a similar genetic background.

**Figure 1:**
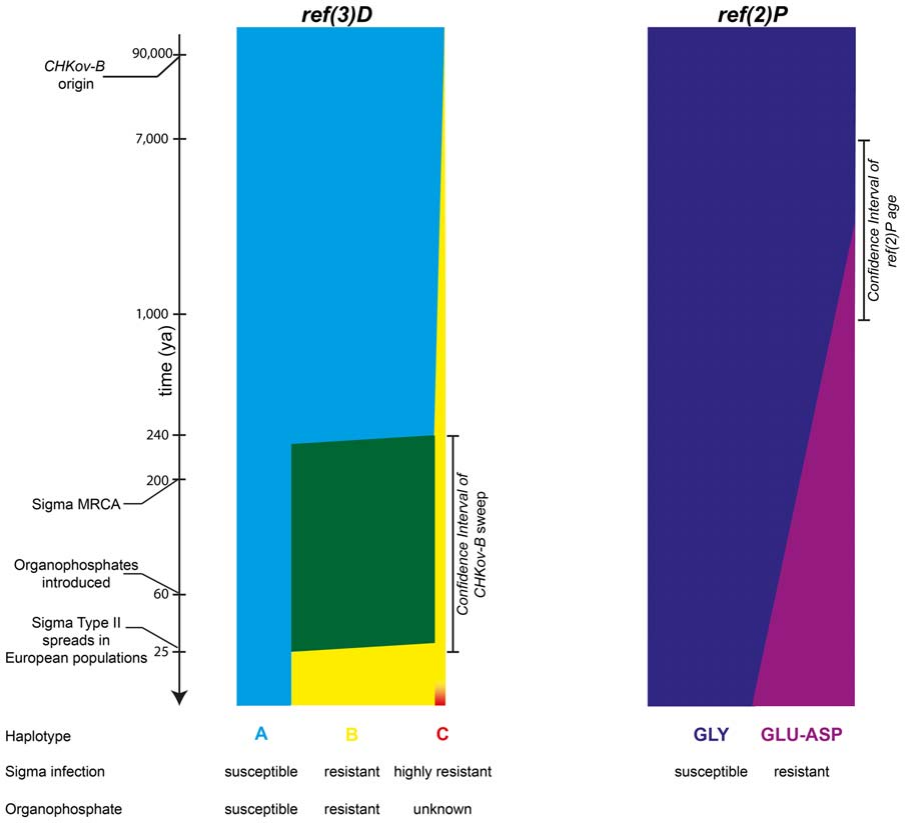
The timeline of putative selective events at two loci with alleles refractory to sigma virus infection. At the *ref(3)D* locus, a selective sweep occurred between 25 and 240 years ago (indicated in green), reducing the frequency of the susceptible A allele (shown in blue) and increasing the frequency of the resistant B allele (yellow) to over 80%. The first unique features of the B allele have been dated to 90,000 years ago. The highly resistant C allele (red) is present in only one of the lines tested. At the *ref(2)P* locus, the GLU-ASP resistance allele is present at about 20% frequency in some contemporary populations, and is believed to have arisen 1,000–7,000 years ago.

The new study of Magwire et al. [3] links another adaptive phenotype, viral resistance, to the *CHKov* genes. The sigma virus has been found to infect up to 20% of *D. melanogaster* flies in field populations. At least six separate genes that reduce infection rates have been mapped in *D. melanogaster* [7]. Sigma-resistant alleles of the *ref(2)P* locus of *D. melanogaster* have previously been characterized and display patterns of polymorphism consistent with a selective sweep [8]. Magwire et al. [3] used a positional cloning approach involving some of the genetic tools available for *D. melanogaster* to molecularly characterize the second of the six genes, *ref(3)D*. The resistant mutation involves a complex rearrangement of the *CHKov1* and *CHKov2* genes, with gene duplications derived from the allele originally characterized by Aminetzach et al. [6]. Thus the naturally occurring allelic series involves three alleles: the ancestral allele that is purportedly susceptible to an OP insecticide and the sigma virus, the *DOC* insertion allele characterized as resistant to an OP and moderately resistant to sigma viruses, and a derived, highly virus-resistant allele (alleles A, B, and C, respectively, in Figure 1).

As in the case of *Cyp6g1*, it appears that the next step in an allelic series has arisen before the previous step has swept to fixation. What is the significance of this? We might expect that in a species with high population substructure, independent alleles may arise and compete against each other depending on the degree of gene flow. However, *D. melanogaster* populations are not thought of as highly structured and the fact the alleles in an allelic series are not independent, but are nested, indicates that *D. melanogaster* populations are large enough to increase the probability of subsequent mutation, even while the previous allele is at a low to moderate frequency.

On the other hand, these results suggest mutation may still be limiting. The most adaptive allele at a gene may be two, three, or more mutational steps away. This may be because the initial adaptive allele is negatively correlated with other important traits, while the subsequent alleles ameliorate these trade offs or costs. Alternatively, the allelic series may reflect a “Red Queen” phenomenon, where a molecular arms race between host and pathogen means that new alleles must arise in the host species, to counter the new alleles in the pathogen species. In that case, it is not that the organism starts multiple steps away from the “adaptive peak”, but that after each step, the “adaptive landscape” changes.

Magwire et al. [3] suggest that the adaptive response to the sigma virus has pre-adapted *D. melanogaster* to OP insecticides. However, it is now unclear how important OPs have been to selection at this locus. One of the mysteries about the *DOC* element insertion into the *CHKov1* gene is that the age of the allele (estimated to be ∼90,000 years), as determined by its divergence from the ancestral allele, is much older than the use of insecticides and the age of the selective sweep, which was determined from the patterns in nearby polymorphisms. The presence/absence of the *DOC* insertion is also correlated with the presence/absence of seven amino acid changes affecting a predicted protein that is substantially shortened and altered relative to that encoded by the ancestral allele. Now we know of another selective agent, namely the sigma virus, that is thought to have been infecting *D. melanogaster* for at least 200 years, but probably longer [9]. A highly virulent variant of the sigma virus is thought to have spread through European *Drosophila* populations in the 1980s, and that is possibly responsible for the recent sweep [10].

Magwire et al.'s [3] findings should motivate molecular and biochemical investigations of the various alleles of *CHKov1* and *2*, and of the somewhat mysterious group of paralogous proteins currently dubbed “choline kinase–like”. Finally, such examples of allelic series not only tell us about population size and structure, but also provide important empirical examples of how fast adaptive evolution at a single locus can be, and should motivate the search for other “adaptive allelic series” that will help us understand the limits and dynamics of adaptation.
